# Gut Microorganisms as Markers of Hyperandrogenemia in Premenopausal Women with Polycystic Ovary Syndrome

**DOI:** 10.3390/ijms27072974

**Published:** 2026-03-25

**Authors:** Larisa Suturina, Natalia Belkova, Tuyana Sidorova, Nadezhda Smurova, Ilia Igumnov, Lyudmila Lazareva, Irina Danusevich, Iana Nadeliaeva, Leonid Sholokhov, Liliia Belenkaia, Alina Atalyan

**Affiliations:** Federal State Budgetary Scientific Institution “Scientific Centre for Family Health and Human Reproduction Problems”, 16, Timiryazeva Str., 664003 Irkutsk, Russia; nlbelkova@gmail.com (N.B.); sidorovat@sbamsr.irk.ru (T.S.); nadinasmurova@mail.ru (N.S.); ilya.igumnov.86@bk.ru (I.I.); lirken_@mail.ru (L.L.); irinaemails@gmail.com (I.D.); ianadoc@mail.ru (I.N.); lfshol@mail.ru (L.S.); drblv@mail.ru (L.B.); alinaa@mail.ru (A.A.)

**Keywords:** androgens, hyperandrogenemia, polycystic ovary syndrome, PCOS, gut microbiota, amplicon metasequencing

## Abstract

Previously, the role of decreased biodiversity of gut microbiota in polycystic ovary syndrome (PCOS) was demonstrated, but the objective criteria for assessing the representation of microorganisms associated with hyperandrogenemia (HA) were limited. A total of 175 premenopausal women (26 women with PCOS and HA and 149 women without HA, including 19 healthy controls) were recruited during the Eastern Siberia PCOS Epidemiology and Phenotype (ESPEP) Study (2016–2019). Methods included a questionnaire survey, clinical examination, pelvic U/S, blood and feces sampling. Gut microbiome was analyzed by high-throughput sequencing of the V1–V3 of the variable regions of the 16S rRNA gene (Illumina MiSeq, San Diego, CA, USA). Amplicon libraries of 16S rDNA were processed using the QIIME2 bioinformatics pipeline. All data were analyzed using R 3.6.3. The gut microbiocenosis in women with HA was characterized by a higher representation of *Lactobacillus* and a lower prevalence of the Clostridia class. For *Faecalibacterium*, *Christensenellaceae_R-7_group*, and *[Eubacterium] eligens group* the cut-off values of their relative presence, associated with HA, were estimated as: ≤0.043%, ≤0.039%, and ≤0.02%, respectively. Conclusions: Women with PCOS-associated HA demonstrate a lower prevalence, predominantly, of Clostridia class gut microorganisms, compared with those without any forms of HA. The study presents the quantitative criteria for assessing the representation of gut microorganisms, negatively associated with hyperandrogenic phenotypes of PCOS. The threshold values proposed may be useful to justify the administration of probiotics in PCOS patients with HA.

## 1. Introduction

Polycystic ovary syndrome (PCOS) is the most frequent cause of hyperandrogenic disorders in premenopausal women. In turn, hyperandrogenemia (HA), or so-called biochemical hyperandrogenism, is one of the leading manifestations of PCOS, associated with metabolic syndrome and increased cardiovascular risk [1,2]. Currently, the role of gut microbiota in the pathogenesis of PCOS, as well as the relationship between microbiome and cardio-metabolic disorders, has become the subject of active study [3,4,5,6,7,8,9,10].

A pilot study of the gut microbiome in patients with PCOS revealed lower diversity and altered phylogenetic composition compared to the control group [3]. In this study, the authors did not find significant differences in any taxa with a relative abundance >1%. However, when assessing the indicators of rare taxa, the relative abundance of some of them was significantly lower and associated with reproductive parameters in patients with PCOS. These authors described such taxa as bacteria from order ML615J-28 (phylum Mycoplasmatota) and family S24-7 (phylum Bacteroidota) [3].

In the study of the association of microbiota with clinical parameters [4], in which women with PCOS were analyzed taking into account body mass index (BMI), the authors showed significant differences in the species composition of the gut microbiota between women with PCOS and a non-obese control group. In this study, bacterial taxa increased in abundance in PCOS, including *Bacteroides*, *Escherichia*/*Shigella*, and *Streptococcus*, which in turn were positively correlated with testosterone and BMI. Furthermore, taxa decreased in abundance in PCOS (*Akkermansia* and Ruminococcaceae) showed opposite relationships with body weight and sex hormones.

Available data on gut microbiota biodiversity primarily support a decrease in the values of at least one of the alpha diversity indices in PCOS, while the scientific findings regarding the diversity differences between non-androgenic and hyperandrogenic women are contradictory [3,5,11,12,13,14,15,16,17,18,19,20,21]. In a study of gut microbiota in women with PCOS and HA [5], regression analysis revealed that total testosterone negatively correlated with bacterial diversity. The relative abundance of eight bacterial taxa differed significantly between the gut microbiota of healthy women and women with PCOS. These bacteria included *Porphyromonas*, *Bacteroides coprophilus*, *Blautia*, *Faecalibacterium prausnitzii*, *Anaerococcus*, *Odoribacter*, *Roseburia*, and *Ruminococcus bromii*. When examining differences in the gut microbiota between normal-weight women with PCOS and women with PCOS and insulin resistance (IR), no significant differences in alpha- and beta-diversity were observed between the groups [6].

In another study, the authors noted an increase in the relative abundance of representatives of the family Bacteroidaceae in the PCOS and IR groups and a decrease in the abundance of the family Prevotellaceae [12]. Using correlation analysis, they showed that elevated levels of clinical parameters, including insulin resistance, sex hormones, and inflammation, correlated positively with the abundance of Bacteroidaceae, but negatively with the abundance of Prevotellaceae.

It was also shown that several species of microorganisms, including *Parabacteroides merdae*, *Bacteroides fragilis*, as well as *Escherichia* and *Shigella*, were statistically significantly more common in the group of patients with PCOS, whereas an increase in the number of *Faecalibacterium prausnitzii* was observed in the control group [13]. A deficiency of these bacteria may lead to changes in the production of short-chain-free fatty acids, which may affect the integrity of the intestinal barrier. In fact, Gram-negative bacteria are known to produce lipopolysaccharides, which can cause inflammation, insulin resistance, and obesity when they enter the bloodstream. It is noteworthy that R. Liu et al. demonstrated that the number of some Gram-negative bacteria belonging to the genera *Bacteroides* and *Escherichia*/*Shigella* was significantly higher in the gut microbiota of patients with PCOS [4]. X. Qi et al. reported that *Bacteroides vulgatus* levels were significantly elevated in the gut microbiota of PCOS patients, accompanied by decreased levels of glycodeoxycholic and tauroursodeoxycholic acids [22]. The same study also noted that bile acid metabolism is one of the most important metabolic pathways affected by changes in the gut microbiota in PCOS patients.

In general, while noting the presence of more pronounced gut microbiota dysbiosis in patients with PCOS, various authors identify various bacterial taxa that correlate with clinical parameters and HA associated with PCOS.

Hereby, some bacterial species are considered markers of gut dysbiosis associated with PCOS [14,22,23,24,25,26,27,28,29]. At the same time, the different groups of authors consider the role of the same bacterial species controversial [14,22,23,24,25,26,27,28,29,30]. Moreover, there is a significant lack of information on the composition of gut microbiota in PCOS phenotypes characterized by hyperandrogenemia. The majority of studies focus on PCOS in general.

We previously demonstrated a decrease in key diversity indices in hyperandrogenic PCOS phenotypes [18] and proposed cut-off points for the main indices classifying women with or without hyperandrogenemia [20]. Taking into account the published data, it seems important to determine which specific microorganisms are responsible for the decrease in the biodiversity of the gut microbiome in PCOS patients with HA and to elaborate the cut-offs for the relative abundance of gut microorganisms, negatively associated with HA, which could be useful for justifying patients’ management.

Therefore, our study objectives were to create the quantitative criteria for assessing the relative representation of gut microorganisms negatively associated with HA.

## 2. Results

### 2.1. Main Characteristics of Premenopausal Women

The main characteristics of the examined women, depending on the presence or absence of hyperandrogenemia, are presented in Table 1. By anthropometric characteristics, women with hyperandrogenemia did not differ significantly from women in the comparison group in general and healthy women in particular. The distribution of women with HA included in the main group (group 1, *n* = 26) by phenotype was as follows: with phenotype A—11/26 (42.3%), B—6/26 (23.1%), C—9/26 (34.6%), *p* > 0.05. When assessing the main characteristics of PCOS in the groups of women examined, consistently higher values of ovarian volumes and the number of follicles were found in hyperandrogenemia, as well as a significantly higher proportion of women with hirsutism, oligo-ovulation (OA), and polycystic ovarian morphology (PCOM) according to ultrasound (Table 1).

### 2.2. Gut Microbiota in Premenopausal Women with HA

Firstly, we characterized the gut microbiota of the examined women at the phylum, class, and genus taxonomic levels. As shown in Table 2, the representation of the Bacteroidota phylum was statistically significantly higher in the HA group as compared to the controls; whereas the Bacillota phylum was detected significantly less frequently in the HA group than in the control group. At the same time, we showed a statistically significantly lower representation of the Clostridia class in the main group compared to the control group. Quantitative characteristics of the proportions of other represented classes in the gut microbiota of women in the compared groups did not differ significantly. We did not detect Verrucomicrobiota in the gut microbiota of women with HA, whereas this phylum was present, albeit slightly, in the gut of women with the absence of HA. The proportions of the Pseudomonadota, Actinomycetota, Fusobacteriota, Candidatus Melainobacteriota, and Desulfobacterota phyla in the gut microbiota of women in the compared groups did not differ quantitatively.

When assessing the gut microbiocenosis of the examined women at the genera level, in the group with HA vs. women without HA, a statistically significant increase in the relative abundance was noted for *Catenibacterium* and *Lactobacillus* (class Bacilli), and for *Oxalobacter* (class Gammaproteobacteria). At the same time, in the HA group, *Faecalibacterium* and *Ruminococcaceae_Incertae Sedis* (class Clostridia), *Acidaminococcus* (class Negativicutes), *Delftia* (class Gammaproteobacteria) were significantly less represented.

In the control group, a significant increase was found compared to the main group in the relative abundance of *Faecalibacterium*, *Christensenellaceae_R-7_group*, *[Eubacterium] eligens group*, and *Oscillospirales UCG-010* (class Clostridia), as well as *Delftia* (class Gammaproteobacteria). Along with this, in HA, a statistically significantly higher representation of *Catenibacterium* (class Bacilli) was observed compared to the control group.

Then, we assessed the degree of relationships between microbiome characteristics and hormone levels in the total group of examined women. When analyzing associations of gut microbiota at the phylum, class, and genus levels, with serum androgens, no strong associations were found (Table 3).

However, at the phylum level, a weak negative correlation was recorded between Pseudomonadota and DHEAS, and at the class level, a negative association was found between the representation of the class Clostridia, testosterone levels, and FAI. Furthermore, a negative association was found between Gammaproteobacteria and DHEAS.

When assessing the gut microbiota at the genus level, a negative relationship was found between *Faecalibacterium*, *Christensenellaceae_R-7_group*, and *[Eubacterium] eligens group* with FAI, suggesting that these microorganisms may have potential protective effects against HA. *Delftia* and *Ruminococcaceae_Incertae Sedis* had also demonstrated the weak negative associations with FAI, but their quantitative representation in the gut microbiome of the examined women was significantly lower than that of the above-mentioned microorganisms.

Then, we used data from women with PCOS and HA and a comparison group (without HA), and applied ROC analysis to determine threshold values for the relative abundance of gut microorganisms, potentially suitable to support the probiotics administration in PCOS patients (Table 4).

According to the data obtained, if any value is below the established threshold (relative amount of *Faecalibacterium* ≤ 0.043%, *Christensenellaceae_R-7_group* ≤ 0.039%, and *[Eubacterium] eligens group* ≤ 0.002%, this can be considered as a basis for prescribing probiotic drugs to the patient.

## 3. Discussion

Polycystic ovary syndrome (PCOS) is the most common endocrine disorder in women, associated with reproductive disorders and multiple comorbidities. Recently, the relationship between gut microbiota, PCOS and cardio-metabolic comorbidities was reported by many authors [3,4,5,6,7,8,9,10].

Modern molecular diagnostic methods based on high-throughput sequencing (meta-sequencing) of bacterial 16S rRNA genes are currently used not only in research projects and clinical trials but also in genetic laboratories providing services to the public. This methodological approach allows for the qualitative and quantitative identification, according to various estimates, of between 100 and 300 genera and approximately 500 species, including uncultured microorganisms. Analysis of amplicon library meta-sequencing data is of great interest for identifying changes in the relative quantitative composition of the microbiota, which in turn are associated with the characteristics of pathological changes in patients. Assessing not only qualitative but also quantitative changes in the composition of the intestinal microbiome may be a non-invasive way to identify dysbiosis associated with PCOS and hyperandrogenemia in women of reproductive age.

Kirillova E. et al. (2023) [8] demonstrated that the gut microbiota in PCOS is characterized by a decrease in species richness and an altered balance of microbial communities, which is most pronounced in the reduced abundance of symbiotic species of *Clostridium*, *Bacteroides*, and a number of other bacteria that provide intestinal colonization resistance. The authors also reported that there is an increase in the population of opportunistic species of *Clostridium* and *Staphylococcus*, which produce exotoxins associated with chronic subclinical inflammation, excess adipose tissue, and the development of insulin resistance. According to the authors, a high level of intestinal colonization with commensal microorganisms of the *Clostridium leptum* group (>9 Lg GE/g) can be expected to result in higher efficacy of metformin therapy. A drawback of this study is that the authors have not established the role of decreased prevalence of the following microorganisms: *Faecalibacterium*, *Christensenellaceae_R-7_group*, and *[Eubacterium]_eligens_group* in HA in women with PCOS and have not elaborated threshold values for their abundance that are prognostically significant for HA.

Previously, we demonstrated the significant decrease in alpha diversity of the gut microbiome compared with healthy women without any signs of PCOS, and then developed criteria for assessing alpha diversity using cut-off points for the most significant indices, which can be useful for monitoring the results of different therapeutic interventions (prebiotics, probiotics, etc.) in hyperandrogenic phenotypes of PCOS [18,20].

Nevertheless, it was important to find the specific microorganisms that are responsible for the decrease in biodiversity. In the current study, we have established the cut-offs for the relative amount of *Faecalibacterium*, *Christensenellaceae_R-7_group*, and *[Eubacterium]_eligens_group*—the microorganisms negatively correlated with androgens, and less represented in the gut microbiome of women with HA. We consider the obtained data as useful for justifying the prescription of probiotics to women with PCOS and hyperandrogenemia.

Producers of short-chain fatty acids (SCFA), including butyrate producers, actively maintain the gut barrier and its permeability, and their reduced abundance might be associated with increased CVD risk among women with PCOS [31]. A hypothesis called DOGMA (dysbiosis of gut microbiota) with microbiological paradigm in PCOS etiology [32] suggested that gut dysbiosis increased the gut mucosal permeability and lipopolysaccharides (LPS) from opportunistic colonic bacteria enter the blood circulation and contact the cardiomyocytes and cardiac fibroblasts. These patterns lead to an increase in cytokines such as IL-1, IL-6, IL-22, IL1-β, and TNF-α [31,33,34]. It was also shown that IL-1, IL-6, and TNF-α are higher in women with PCOS, thus explaining their increased risk of CVD and the possible protective role of a healthy gut microbial community.

*Faecalibacterium* is one of the major butyrate producers in the gut, and it is significantly depleted not only in women with PCOS [35,36] but also in obesity, cardiovascular pathologies, and diabetes [37,38,39]. Other SCFA producers include representatives of the *Christensenellaceae_R-7_group* and *[Eubacterium]_eligens_group*. A decrease in their quantitative indicators has been determined in various pathologies, both infectious [40] and non-infectious [41,42,43,44,45]. Researchers note different intestinal microbiota profiles in patients who respond and do not respond to treatment [43,44].

Taken together, these results demonstrate that our target taxonomic groups of *Faecalibacterium*, *Christensenellaceae_R-7_group*, and *[Eubacterium]_eligens_group* may be useful biomarkers for HA in PCOS. As a result of our study, we proposed for the first time the cut-offs for a relative abundance of gut microorganisms, negatively associated with HA, and we suggest the potential of microbiome analysis for developing interpretable, noninvasive tools for managing HA in PCOS.

A limitation of our study: (i) the microbiome-associated markers of hyperandrogenemia in premenopausal women with polycystic ovary syndrome were identified during the cross-sectional study, and more prospective studies are needed to investigate the prognostic value of our findings, (ii) the methods did not include an assessment of the participants’ dietary habits.

The methodological strengths of our study: (i) all study materials were obtained from the well phenotyped participants who were recruited from the unselected, medically unbiased population, (ii) the participants with and without HA were comparable by age and BMI, which prevents biases due to the influence of these important co-founders, (iii) we used a highly efficient method (LC-MS/MS) to measure testosterone levels, which is critical for the qualitative assessment of hyperandrogenemia and we also conducted a search on the composition of the gut microbiota, exploring the modern molecular diagnostic methods based on high-throughput sequencing (meta-sequencing) of bacterial 16S rRNA.

## 4. Materials and Methods

We performed this research as a sub-study of cross-sectional, multicenter, institution-based Eastern Siberia PCOS Epidemiology and Phenotype (ESPEP) Study, conducted in the medically unbiased population of premenopausal women living in similar geographic and socioeconomic conditions in the Irkutsk region and the Republic of Buryatia (Eastern Siberia, Russian Federation) from 2016 to 2019 (ClinicalTrials.gov ID: NCT05194384) [46].

For PCOS diagnosis, the Rotterdam Criteria (2003) were used: a presence of any two of three criteria—hyperandrogenism, oligo/anovulation, and polycystic ovarian morphology—with no conditions with similar symptoms (hyperprolactinemia, hypothyroidism, 21-hydroxylase-deficient non-classic congenital adrenal hyperplasia (NC-CAH), premature ovarian failure) [47].

The inclusion and exclusion criteria for the ESPEP study were previously reported [46]: Inclusion criteria were (1) premenopausal women aged 18 to 44 years, (2) providing written, informed consent, (3) compliance with all study procedures and available for the duration of the study, and (4) all races and ethnicities. Exclusion criteria were unwillingness to participate and/or absence of compliance with all study procedures and requirements, current pregnancy or lactation, history of surgery, and current or previous (within 3 months) intake of hormonal medications and insulin sensitizers).

For our sub-study, we also excluded women with antibiotic intake within one month before the recruitment.

The inclusion criteria for the main group (Group 1) of our sub-study were as follows: premenopausal participants of the ESPEP study diagnosed with PCOS and HA associated with phenotypes A, B, and C, according to Rotterdam criteria (2003), who were willing to provide stool samples and take part in this sub-study. Women with PCOS without HA and participants with HA who were not diagnosed with PCOS were not included in Group 1.

The inclusion criteria for Group 2 were: premenopausal women who participated in the ESPEP Study, without HA, who were willing to provide stool samples and take part in this sub-study. Patients with hyperprolactinemia, hypothyroidism, 21-hydroxylase-deficient non-classic congenital adrenal hyperplasia (NC-CAH), and premature ovarian failure) were not included in this group, as well as in Group 1.

Among the ESPEP Study participants who completed the evaluation for PCOS, after exclusion of related disorders, a total of 994 women were considered for including to our study (176 with HA, including 81 PCOS patients, and 718 women without HA, including 84 PCOS patients).

After assessing the inclusion and exclusion criteria, a total of 175 premenopausal women (26 women with PCOS and HA—Group 1, and 149 women without HA (Group 2)) were recruited in our study. The group 2 included 37 women with PCOS who had not HA. Thus, the main criterion for dividing into groups was the presence or absence of HA.

Among the women without HA (Group 2), we formed the Control group—a subset of clinically healthy women (*n* = 19). Inclusion criteria for the Control group were as follows: the absence of signs of PCOS, regular menstrual cycles (21–35 days), modified Ferriman–Gallwey score (mF-G) < 3, absence of alopecia and acne, ovarian volume < 10 cm^3^, and follicle number per ovary (FNPO) < 12. Exclusion criteria for controls were: history of chronic diseases, BMI < 18 or ≥30 kg/m^2^, elevated blood pressure, or abnormal levels of androgens, fasting glucose, prolactin (PRL), follicle-stimulating hormone (FSH), thyroid-stimulating hormone (TSH), or 17-hydroxyprogesterone.

Methods included a questionnaire survey, anthropometry with body mass index (BMI) calculation (weight (kg)/height (m^2^)), vital signs, modified Ferriman—Gallwey (mF-G) scoring, gynecological examination, lab tests, and pelvic ultrasound (US). Pelvic US was performed using the portable ultrasound scanner Mindray M7 (Mindray Bio-Medical Electronics Co., Shenzhen, China) with a transvaginal (5.0–8.0 MHz) and transabdominal (2.5–5.0 MHz) probe.

### 4.1. Laboratory Methods

#### Androgen Assessment

Blood serum total testosterone (TT) was measured using liquid chromatography–mass spectrometry (LC-MS/MS, Shimadzu LCMS-8060, Shimadzu, Kioto, Japan). Sex hormone–binding globulin (SHBG) was measured using ELISA Alkor-Bio test kits (Alkor-bio, Saint Petersburg, Russia) and Elx808 microplate photometer (Bio-Tek Instruments, Winooski, VT, USA). The free androgen index (FAI) was calculated as: FAI = (TT/SHBG) × 100. Dehydroepiandrosterone-sulfate (DHEAS) was assessed using immunochemiluminescence kits and Immulite 1000 analyzer (Siemens Health Care Diagnostics Inc., Flanders, NJ, USA).

Hyperandrogenemia was defined by elevation above the normal reference ranges for at least one of: TT, FAI, or DHEAS, as required by the current guidelines [47]. The upper normal levels (UNL) for androgens were previously determined from the 98th percentiles for these parameters in the healthy controls and reported [48]. The UNLs for TT and FAI varied by ethnicity in our healthy controls: 73.9 ng/dL (2.56 nmol/L) and 6.9 for Caucasians; 41 ng/dL (1.42 nmol/L) and 2.9 for Asians and women of mixed (Caucasian/Asian) ethnicity, respectively. For DHEAS, UNLs were similar for all races: 355 μg/dL.

### 4.2. Other Hormonal Methods

TSH, LH, FSH, prolactin (PRL) and 17-OH progesterone were determined using competitive solid-phase ELISA test kits (Alkor-Bio, Saint Petersburg, Russia) on the Elx808 analyzer (Bio-Tek Instruments, Winooski, VT, USA). Anti-Müllerian hormone (AMH) was measured using Beckman Coulter kits on ELx808 (Bio-Tek Instruments, Winooski, VT, USA).

### 4.3. Methods for Gut Microbiota Studying

Fecal sampling, genomic DNA isolation and high-throughput sequencing of the V1–V3 variable regions of the 16S rRNA gene were described previously [18]. Amplicon libraries of 16S rDNA (Bioproject PRJNA899143) were processed using the QIIME2 v.2022.11 bioinformatics pipeline to conduct a comparative metagenomic study [49]. Amplicon sequencing variants (ASV) were generated using the DADA2 v.2022.11.1 algorithm, which allows detection, correction, and filtering of amplicon errors and chimeric sequences [50]. DADA2 parameters for trimming paired-ended reads were as follows: for forward reads, trim 20, trunc 260; for reverse reads, trim 20, trunc 260. The resulting representative sequences were used to determine their taxonomic classification using the sklearn-based Naive Bayes classifier trained on the SILVA v.138 with 99% 16S rDNA full-length database [51].

### 4.4. Statistical Analysis

Sample size calculations for the total population were based on the following formula: *n* = [(Z2 1–α p(1 − p)]/D2, where *n*—individual sample size, Z1–α = 1.96 (when α = 0.05), p—assumed PCOS prevalence according to the previously published data, and D—absolute error.

The data were collected using Research Electronic Data Capture (REDCap) [52]. Outliers were identified during the Exploratory Data Analysis using the box-plot and 3σ methods. Managing missing data: In our research dataset, there were two types of missing data—missing completely at random (MCAR) and missing at random (MAR). We recorded all missing values with labels of “N/A” to make them consistent throughout our dataset. Pairwise deletion was used when the dataset was analyzed. To estimate the assumption of the normal distribution of our datasets, we performed a formal statistical test—the Shapiro–Wilk test. Chi-square (χ^2^) was used for frequency data. We used a Student’s t-test to compare the mean values of the data with an independent sample, which followed a normal distribution, or a Mann–Whitney U-test to compare the ratio between two groups in another case. A correlation analysis was conducted with the calculation of the nonparametric Spearman rank correlation coefficient -r. The strength of the relationship was assessed as follows: 0.01 ≤ r ≤ 0.29—weak relationship; 0.3 ≤ r ≤ 0.69—moderate relationship; 0.7 ≤ r ≤ 1.00—strong relationship between the parameters. The statistical significance was established at *p* < 0.05. Statistical analysis included ROC analysis for cutoffs development. All data were analyzed using R 3.6.3 (a free software environment for statistical computing and graphics).

## 5. Conclusions

Hyperandrogenemia is a common endocrine disorder, and PCOS is its main cause in women of reproductive age. The previously established association of PCOS with gut dysbiosis justified the need to clarify which specific microbiome imbalances are particularly significant in hyperandrogenemia, as data were sparse and research findings contradictory.

The results of our study have shown that women with PCOS-associated HA demonstrate a statistically significant increase in the representation of class Bacilli in the gut microbiome and a lower prevalence of Clostridia class gut microorganisms, compared with those without any forms of HA.

The proposed cut-off values for the relative abundance of marker microorganisms, which decrease is associated with hyperandrogenemia, may be useful to justify the probiotics administration and monitor the effectiveness of treatment in PCOS patients.

However, prospective studies are needed to test the effectiveness of the proposed approach to assessing the state of the gut microbiome in relation to hyperandrogenic disorders.

## Figures and Tables

**Table 1:** Main characteristics of premenopausal PCOS patients with HA, participants without HA, and in the control group.

Parameter	Group 1 (*n* = 26) 1	Group 2 (*n* = 149) 2	Controls (*n* = 19) 3	*p* ^#^
M ± SD Me (IQR)				
Age, years	30.8 ± 5.6 32 (27; 35)	33.5 ± 6.2 34 (30; 39)	34.3 ± 4.3 35 (31.5; 38.5)	0.05 ^1–2^ 0.04 ^1–3^
Height, cm	163 ± 8.2165 (158; 169)	164 ± 6.0165 (160; 168)	163 ± 6.0160 (158; 166)	0.62 ^1–2^ 0.78 ^1–3^
Weight, kg	71.5 ± 17.0 68.4 (62.8; 78.5)	71.4 ± 15.068.7 (60.5; 78.8)	65.7 ± 12.5 65.7 (53.4; 74.0)	0.96 ^1–2^ 0.27 ^1–3^
BMI, kg/m^2^	26.9 ± 6.125.9 (22.4; 31.3)	26.4 ± 5.425.8 (22.0; 29.0)	24.6 ± 3.525.1 (21.3; 27.4)	0.70 ^1–2^ 0.22 ^1–3^
WC, cm	80.7 ± 13.576.5 (72.5; 89.5)	79.6 ± 12.778.0 (70.0; 86.0)	76.0 ± 8.976.0 (68.5; 82.5)	0.76 ^1–2^ 0.61 ^1–3^
SBP, mm Hg	125 ± 14.0125 (115; 133)	124 ± 13.2124 (113; 132)	117 ± 11.7114 (110; 124)	0.64 ^1–2^ 0.05 ^1–3^
Right ovary volume, cm^3^ *	11.6 ± 5.0 10.8 (9.1; 13.5)	10.8 ± 10.9 8.5 (5.9; 11.7)	6.4 ± 1.7 6.1 (5.0; 7.3)	0.016 ^1–2^ 0.000 ^1–3^
Left ovary volume, cm^3^	8.6 ± 3.3 8.6 (6.7; 9.5)	9.4 ± 8.5 7.4 (5.5; 10.6)	6.2 ± 2.0 6.4 (4.7; 7.2)	0.486 ^1–2^ 0.009 ^1–3^
FNPO, right ovary	11.4 ± 3.8 12.0 (9.0; 14.0)	9.1 ± 4.3 8.0 (6.0; 12.0)	6.6 ± 3.1 6.0 (5.0; 7.5)	0.006 ^1–2^ 0.000 ^1–3^
FNPO, left ovary	10.6 ± 3.2 12.0 (8.0; 13.0)	8.4 ± 4.0 7.0 (5.0; 12.0)	6.5 ± 2.5 6.0 (5.0; 8.0)	0.003 ^1–2^ 0.000 ^1–3^
LH, mIU/mL	13.0 ± 11.2 10.0 (6.3; 15.5)	7.6 ± 7.4 5.6 (3.2; 9.2)	6.5 ± 5.2 5.8 (3.9; 6.9)	0.00 ^1–2^ 0.00 ^1–3^
FSH, mIU/mL	5.9 ± 1.8 5.9 (5.0; 7.2)	5.8 ± 5.4 5.1 (3.7; 6.4)	5.7 ± 1.8 6.1 (4.4; 6.8)	0.04 ^1–2^ 0.79 ^1–3^
PRL, mIU/mL	322 ± 161 291 (233; 435)	336 ± 189 286 (215; 408)	296 ± 132 248 (189; 418)	0.95 ^1–2^ 0.73 ^1–3^
TSH, IU/L	1.6 ± 0.8 1.6 (0.9; 1.9)	1.8 ± 1.6 1.5 (1.1; 1.9)	1.5 ± 0.7 1.6 (1.0; 1.9)	0.76 ^1–2^ 0.94 ^1–3^
AMH, ng/mL	6.8 ± 5.8 4.6 (2.5; 8.6)	4.5 ± 5.0 2.7 (1.0; 6.1)	2.8 ± 2.1 2.0 (1.3; 3.6)	0.01 ^1–2^ 0.00 ^1–3^
17OHP, nmol/L	5.4 ± 3.2 4.9 (2.7; 7.5)	5.3 ± 3.5 5.0 (2.6; 7.3)	3.9 ± 2.8 3.3 (2.0; 5.6)	0.72 ^1–2^ 0.11 ^1–3^
TT, ng/dL	62.8 ± 28.8 55.6 (44.5; 81.1)	27.7 ± 14.7 26 (17.9; 36.5)	23.5 ± 11.8 25.6 (16.2; 28.4)	<0.001 ^1–2^ <0.001 ^1–3^
SHBG, nmol/L	65.3 ± 52.9 40.3 (31.3; 89.8)	78.9 ± 51.3 69.7 (43.1; 99.3)	89.1 ± 46.9 68.7 (59.3; 108)	0.032 ^1–2^ 0.018 ^1–3^
FAI	5.3 ± 3.8 4.6 (2.2; 6.7)	1.6 ± 1.2 1.3 (0.8; 2.2)	1.1 ± 0.8 1.0 (0.5; 1.4)	0.00 ^1–2^ 0.00 ^1–3^
DHEAS, μg/dL	274 ± 144 213 (151; 389)	172.9 ± 71,8 168 (117; 222)	173.5 ± 65,9 186 (122; 208)	0.00 ^1–2^ 0.04 ^1–3^
*n*/*N* (%)				*p* ^##^
PCOS	26/26 (100.0%)	37/149 (24.8%) *	0/19 (0.0%)	0.000 ^1–3^ 0.000 ^1–2^
HA	26/26 (100.0%)	0/149 (0.0%)	0/19 (0.0%)	0.000 ^1–2^ 0.000 ^1–3^
Hirsutism	10/26 (38.5%)	19/149 (12.7%)	0/19 (0.0%)	0.003 ^1–2^ 0.002 ^1–3^
OA	17/26 (65.4%)	49/149 (32.9%)	1/19 (5.3%)	0.004 ^1–2^ 0.000 ^1–3^
PCOM	20/26 (76.9%)	67/149 (45.0%)	3/19 (15.8%)	0.002 ^1–2^ 0.000 ^1–3^

^#^—Mann–Whitney U Test; ^##^—Chi-square (χ^2^) test; *—PCOS without HA (phenotype D). Abbreviations: BMI is body mass index, WC is waist circumference, SBP—systolic blood pressure, FNPO is follicle number per ovary, LH—luteinizing hormone, FSH—follicle-stimulating hormone, PR—prolactin, TSH—thyroid-stimulating hormone, AMH—anti-Mullerian hormone, 17OHP—17-hydroxyprogesterone, TT—total testosterone, SHBG—sex-hormone-binding globulin, FAI—free androgen index, DHEAS—dehydroepiandrosterone sulfate, PCOS—polycystic ovary syndrome, HA—hyperandrogenemia, OA—olygo/anovulation, and PCOM—polycystic ovarian morphology.

**Table 2:** Gut microbiota composition in premenopausal PCOS patients with HA, participants without HA and in the control group.

Taxon Name/Taxonomic Level	Group 1 (*n* = 26) 1	Group 2 (*n* = 149) 2	Controls (*n* = 19) 3	*p* ^#^
M ± SD Me (IQR)				
Bacteroidota/phylum	42.7 ± 24.7	36.3 ± 24.4	26.7 ± 17.0	0.23 ^1–2^
	41.7 (25.0; 66.4)	36.8 (12.6; 56.7)	28.3 (12.1; 36.6)	0.04 ^1–3^
Bacillota/phylum	52.8 ± 25.6	58.4 ± 26.1	70.2 ± 18.7	0.29 ^1–2^
	50.9 (31.4; 73.4)	56.7 (37.0; 83.4)	69.8 (59.7; 85.3)	0.03 ^1–3^
Clostridia/class	42.0 ± 22.0	49.9 ± 25.5	63.1 ± 18.3	0.14 ^1–2^
	32.7 (27.5; 55.7)	47.1 (28.2; 69.8)	64.1 (50.5; 80.7)	0.00 ^1–3^
*Catenibacterium*/genus	0.05 ± 0.1	0.02 ± 0.08	0.01 ± 0.03	0.02 ^1–2^
	0.0 (0.0; 0.03)	0.0 (0.0; 0.00)	0.0 (0.0; 0.00)	0.04 ^1–3^
*Faecalibacterium*/genus	0.07 ± 0.12	0.13 ± 0.17	0.23 ± 0.23	0.03 ^1–2^
	0.03 (0.01; 0.05)	0.06 (0.02; 0.18)	0.14 (0.07; 0.30)	<0.001^1–3^
*Christensenellaceae_R-7_group*/genus	0.01 ± 0.01	0.03 ± 0.06	0.04 ± 0.06	0.28 ^1–2^
	0.00 (0.00; 0.01)	0.01 (0.00; 0.03)	0.02 (0.00; 0.07)	0.03 ^1–3^
*Lactobacillus*/genus	0.01 ± 0.04	0.00 ± 0.01	0.001 ± 0.004	0.01 ^1–2^
	0.0 (0.0; 0.00)	0.0 (0.0; 0.0)	0.0 (0.0; 0.0)	0.15 ^1–3^
*[Eubacterium] eligens group*/genus	0.003 ± 0.01	0.004 ± 0.01	0.007 ± 0.02	0.21 ^1–2^
	0.0 (0.0; 0.00)	0.0 (0.0; 0.004)	0.00 (0.0; 0.01)	0.02 ^1–3^
*Oscillospirales UCG-010*/genus	0.00 ± 0.00	0.0031 ± 0.01	0.01 ± 0.01	0.77 ^1–2^
	0.0007 (0.0; 0.003)	0.001 (0.0; 0.0043)	0.004 (0.0004; 0.00898)	0.04 ^1–3^
*Acidaminococcus*/genus	0.00076 ± 0.001	0.0012 ± 0.00604	2 × 10^−5^ ± 7 × 10^−5^	0.04 ^1–2^
	0.0 (0.0; 0.001)	0.0 (0.0; 0.0)	0.0 (0.0; 0.0)	0.19 ^1–3^
*Delftia*/genus	0.0001 ± 0.00034	0.0004 ± 0.00172	0.0002 ± 0.00027	0.04 ^1–2^
	0.0 (0.0; 0.0)	0.0 (0.0; 0.00026)	0.0 (0.0; 0.00046)	0.02 ^1–3^
*Ruminococcaceae_Incertae Sedis*/genus	0.00021 ± 0.00085	0.0005 ± 0.00144	0.00026 ± 0.00053	0.04 ^1–2^
	0.0 (0.0; 0.0)	0.0 (0.0; 0.00041)	0.0 (0.0; 0.00015)	0.40 ^1–3^
*Oxalobacter*/genus	0.00057 ± 0.00098	0.00029 ± 0.00094	2 × 10^−5^ ± 9 × 10^−5^	0.02 ^1–2^
	0.0 (0.0; 0.00073)	0.0 (0.0; 0.0)	0.0 (0.0; 0.0)	0.02 ^1–3^

^#^—Mann–Whitney U Test.

**Table 3:** Statistically significant correlations of representatives of the gut microbiocenosis with hormones in the examined women (*n* = 175).

Parameters	*r*_S_	*p*
Phylum/hormones		
Bacteroidota and AMH	0.23	<0.001
Bacillota and AMH	–0.22	<0.001
Pseudomonadota and DHEAS	–0.16	0.03
Actinomycetota and AMH	–0.2	0.01
Candidatus Melainobacteriota and FSH	–0.17	0.03
Class/hormones		
Clostridia and TT	–0.15	0.05
Clostridia and FAI	–0.15	0.04
Clostridia and AMH	–0.31	<0.001
Negativicutes and AMH	0.15	0.05
Gammaproteobacteria and DHEAS	–0.15	0.05
Alphaproteobacteria and SHBG	–0.17	0.03
Coriobacteriia and AMH	–0.19	0.01
Actinobacteria and AMH	–0.16	0.04
Genus/hormones		
*Faecalibacterium* and FAI	–0.16	0.03
*Christensenellaceae_R-7_group* and FAI	–0.16	0.03
*[Eubacterium] eligens group* and TT	–0.16	0.03
*[Eubacterium] eligens group* and FAI	–0.17	0.02
*[Eubacterium] eligens group* and AMH	–0.23	<0.001
*Delftia* and FAI	–0.21	0.01
*Ruminococcaceae_Incertae Sedis* and FAI	–0.16	0.03
*Oxalobacter* and AMH	0.2	0.01

**Table 4:** Cut-off values for the relative abundance of significant, as HA markers, microorganisms in the gut microbiocenosis.

Microorganism	Cut-Off (95% CI)	AUC (95% CI)	Sensitivity (95% CI)	Specificity (95% CI)
*Faecalibacterium*	0.043	0.631	0.604	0.731
	(0.043; 0.043)	(0.520; 0.743)	(0.523; 0.685)	(0.538; 0.885)
*Christensenellaceae_R-7_group*	0.039	0.566	0.215	1.000
	(0.039; 0.039)	(0.459; 0.674)	(0.154; 0.282)	(1.000; 1.000)
*[Eubacterium] eligens group*	0.002	0.577	0.356	0.846
	(0.002; 0.002)	(0.477; 0.677)	(0.282; 0.430)	(0.692; 0.962)

Abbreviations: AUC is Area under the curve, CI -Confidence intervals.

## Data Availability

The data presented in this study are openly available in [NCBI] [https://www.ncbi.nlm.nih.gov/bioproject/?term=PRJNA899143] [PRJNA899143] (accessed on 22 March 2026).

## Abbreviations

The following abbreviations are used in this manuscript:

AMH	anti-Müllerian hormone
BMI	body mass index
DHEAS	dehydroepiandrosterone sulfate
DOGMA	dysbiosis of gut microbiota
ESPEP	Eastern Siberia PCOS Epidemiology and Phenotype Study
FAI	free androgen index
FNPO	follicle number per ovary
FSH	follicle-stimulating hormone
HA	hyperandrogenemia
LC-MS/MS	liquid chromatography–mass spectrometry
LH	luteinizing hormone
LPS	lipopolysaccharides
MCAR	missing completely at random
MAR	missing at random
mF-G	Ferriman–Gallwey score
NC-CAH	non-classic congenital adrenal hyperplasia
OA	oligoovulation
PCOS	polycystic ovary syndrome
PCOM	polycystic ovarian morphology
PRL	prolactin
REDCap	Research Electronic Data Capture
SCFA	short-chain fatty acids
SBP	systolic blood pressure
SHBG	sex-hormone-binding globulin
TSH	thyroid-stimulating hormone
TT	total testosterone
UNL	upper normal levels
U/S	ultrasonography
WC	waist circumference
17OHP	17-hydroxyprogesteron
